# Digital Finance and Green Development: Characteristics, Mechanisms, and Empirical Evidences

**DOI:** 10.3390/ijerph192416940

**Published:** 2022-12-16

**Authors:** Rulong Zhuang, Kena Mi, Menglu Zhi, Chaoyang Zhang

**Affiliations:** 1 School of Business, Ningbo University, Ningbo 315211, China; 2 School of International Trade & Economics, Ningbo University of Finance & Economics, Ningbo 315175, China

**Keywords:** digital finance, green development, energy structure, industrial upgrading, technological progress, China

## Abstract

As the emergence of digital finance is relatively short, research results on digital finance mainly focus on products, services, coverage, policies, etc. The mechanism and role of digital finance in influencing green development are still lacking attention. In the above context, this paper used spatial analysis methods to describe spatiotemporal characteristics in detail, and empirically tested the mechanism and path of digital finance affecting green development through spatial econometric models and intermediary models. The results showed that: (1) During the study period, digital finance and green development have been improved to varying degrees, but the inter-provincial differences are still obvious. (2) The spatial trends of digital finance and green development are similar, and the overall performance is “high in the east, low in the west, high in the south, and low in the north”. (3) The empirical tests found that digital finance is an effective force to reduce energy consumption per unit of GDP and improve the level of green development. It validates Hypothesis 1. Meanwhile, the Heterogeneity effect is noteworthy due to different regions, types, and levels. (4) The promotion of green development by digital finance is mainly concentrated in the local region and has not yet shown a significant green spillover effect for surrounding areas. It validates Hypothesis 2. (5) Energy structure, industrial upgrading, and technological progress are three paths for digital finance affecting green development. Hypothesis 3 is verified. Finally, the innovation of this paper lies in the design of the research framework, diversity of research methods, and policy implications. The main contribution is to enrich and expand the environmental finance theory and provide detailed empirical evidence. In addition, we put forward effective measures and suggestions including local governments, financial institutions, and enterprises based on the empirical results. Local governments should pay attention to policy implementation and operation effects, financial institutions constantly need to strengthen the supply of advanced digital financial products and services, and enterprises should attach importance to the use of digital financial tools to achieve green and low-carbon development in the future.

## 1. Introduction

For quite some time, an extensive economic development model with large factor input has caused serious resource and environmental problems, and the concept of green development has become the consensus of the whole society. In 2021, China’s carbon emissions have reached 11.47 billion tons, twice that of the United States (5 billion tons) and four times that of the European Union (2.79 billion tons), and have not yet reached the peak. At the same time, digital finance has developed rapidly. In 2020, China’s mobile payment business and payment finance reached 123.22 billion transactions and 432.16 trillion CNY, with year-on-year growth of 21.48% and 24.50% respectively. The most frequently used mobile payment products are WeChat, Alipay, and UnionPay cloud flash payment. In addition, 150 million users in China have purchased online financial products in 2020. Influenced by the COVID-19 pandemic in 2020, traditional financial services are subject to many restrictions in terms of sales, investment, after-sales, etc., further strengthening the trend of online “contactless” financial services.

Digital finance is a kind of new type of financial service, which is mainly formed by combining the Internet and Information technology with traditional financial services, including mobile payment, online banking, financial service outsourcing, online loans, online insurance, online funds, and other forms. What is important is that digital finance itself is also a kind of green finance. In the process of achieving green development, digital finance will play a non-negligible role in optimizing resource allocation, enhancing technological innovation, and promoting industrial upgrading, and so on. It will become one of the important driving forces to achieve the “dual carbon” goal [1,2,3,4]. The “dual carbon” goal was first proposed by Chinese President Xi at the 75th United Nations General Assembly in 2020. The main contents are that China will improve its national independent contribution, adopt more powerful policies and measures, strive to reach the peak of carbon dioxide emissions by 2030, and strive to achieve carbon neutrality by 2060. Against this background, we also regard the realization of the “dual carbon” goal as the important research content and direction of this paper.

To better achieve the “dual carbon” goal, promote the green transformation of the industry, and reduce carbon emissions, this paper took digital finance as the research object and systematically depicted the spatiotemporal characteristics. On this basis, an econometric model is constructed to judge the impact of digital finance on green development and its path. Compared with the existing research the contribution of this paper mainly lies in the following three points. Firstly, it brings digital finance and green development into the unified research framework, and carefully analyses the mechanism of digital finance affecting green development. Secondly, it deeply explores the specific path of the impact of digital finance on green development and tries to test the mediation effect from the three dimensions: energy structure, industrial upgrading, and technological progress. Thirdly, considering the possible spatial relationship, this paper discusses the spatial effect of digital finance on green development from the perspective of “Local-foreign”. Finally, based on the above research, this paper puts forward countermeasures and enlightenments for promoting the development of digital finance and realizing the goal of green, low-carbon, and sustainable development.

**Figure 1:**
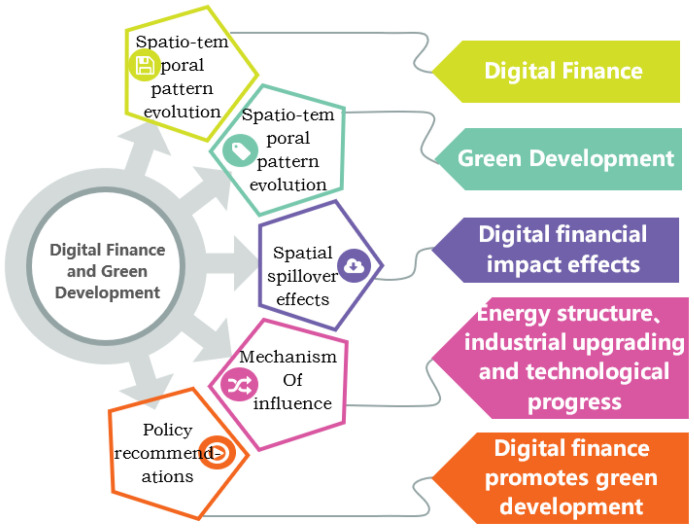
The research framework.

Figure 1 illustrates the research framework of this paper.

## 2. Literature Review

### 2.1. Studies on Digital Finance

With the vigorous development of artificial intelligence, cloud computing, and big data technology, finance has strengthened the comprehensive integration with emerging technologies. Digital finance is emerging in this context. In addition, with the increasingly important role of digital finance, it has gradually become a research hotspot in the financial field [5,6,7]. At present, the academic researches on digital finance mainly focus on two aspects. The first aspect is the discussion of digital finance itself, which originates from a deep understanding of the connotation. These contents include digital financial products, services, industries, supervision, etc. [8,9]. The second aspect is the analysis of the economic and social impact of digital finance through comparison with traditional finance. Those impacts are very extensive, including industrial upgrading, product innovation, technological progress, citizen welfare, poverty alleviation, economic growth, and regional coordinated development, etc. [10,11,12,13,14]. The general conclusion is that digital finance can effectively promote industrial upgrading, technological progress, welfare improvement, and economic development. Some scholars discussed the relationship between government expenditure and intergenerational mobility, which provides a direction for the government to use digital financial tools to play an inclusive role to achieve equal distribution of public services [15]. At the same time, some scholars have also noticed that digital finance will inevitably be affected by various risks while playing a positive role. Therefore, it is indispensable to strengthen financial supervision to ensure its efficient and healthy development [16]. In addition, some scholars pay attention to the impact of e-commerce development closely related to digital finance on consumer behaviors and retail space values and analyzed and explained it through store rents [17]. It is necessary to mention that due to the green nature of digital finance and the proposed carbon emission reduction targets in various countries and regions, the environmental effects of digital finance have also attracted the attention of scholars. These will be explained as follows.

### 2.2. Studies on Green Development

With the continuous development of urbanization and industrialization, the constraints of resources and the environment have gradually become an important obstacle restricting the sustainable development of the economy and society. In this context, the concept of green development has gradually formed and gained consensus at home and abroad [18,19,20]. Green development emphasizes the transformation and optimization of human production and lifestyle, the reduction of resource consumption and environmental damage, and the realization of healthy and sustainable economic and social development. There are many similar concepts to green development, such as sustainable development, green economy, low-carbon economy, green growth, etc. [21,22]. Among them, sustainable development can be seen as the theoretical origin of green development. Through combing the relevant literature at home and abroad, we found that the research on green development mainly includes connotation, concept, level, path, method, etc. Among them, scientific measurement and seeking future development paths have become the focus of many experts and scholars [23]. As one of the important directions of green development, digital finance has attracted more and more attention [24,25]. With the establishment of the carbon trading mechanism, some scholars also began to study the carbon emission reduction effect of the carbon trading mechanism [26]. In addition, due to the impact of the COVID-19 epidemic in recent years, the impact of the COVID-19 epidemic on the stock prices of energy enterprises has also attracted attention [27,28]. Finally, the research on the relationship between economic growth and green development cannot be ignored. The most representative topic is the Kuznets curve [29].

### 2.3. Studies on the Relationship between Digital Finance and Green Development

Digital finance is the integration of traditional finance and modern science and technology, which still has the basic characteristics of traditional finance, so the research on the impact of traditional finance on environmental pollution can provide references for this paper [30]. The relationship between finance and the environment has been studied for a long time in the academic circle, but there has always been controversy. The main academic views can be summarized as follows: The first view is that the prosperity of the financial market contributes to economic growth, but economic growth will also lead to an increase in energy demand, which may eventually lead to more pollutant emissions and increase environmental pressure. The second view is that, in addition to increasing energy demand and pollutant emissions, financial development may also improve energy use and resource allocation efficiency through scientific and technological progress and industrial structure upgrading and may also play a positive role in environmental protection [31,32,33,34]. In addition, the third view holds that there is a nonlinear relationship between financial development and environmental pollution [35]. Based on existing research, this paper attempts to explore the relationship between digital finance and environmental pollution to enrich and expand its scope and contents. On this subject, there is also some relevant research in the academic circle, mainly involving production efficiency, technological innovation, industrial structure, etc. [36,37,38].

Although the above literature provides rich perspectives for our research, there are still some shortcomings. The discussion on digital finance mainly focuses on regulations, services, products, risks, and policies. Regrettably, there is still a lack of enough attention to the internal relationship between digital finance and green development, especially to explore the mechanism of digital finance affecting green development by using reasonable data and scientific methods. In addition, most studies do not consider the possible spatial correlation between digital finance and green development, which will lead to errors in theoretical research and practical analyses.

## 3. Theoretical Analyses and Research Hypotheses

### 3.1. Digital Finance and Green Development

Sustainable development theory and environmental finance theory provide useful guidance for the mechanism analysis of digital finance affecting green development [39,40,41]. From the perspective of sustainable development theory, digital finance has realized its own green transformation through the combination of information technology, reduced the resource consumption of the financial industry itself, and improved operating efficiency. At the same time, compared with traditional finance, digital finance plays a more significant role in supporting and promoting green industry and green technology, and contributes to the sustainable development of the economy and society. The environmental finance theory believes that the environment is a factor that the financial industry needs to focus on. Finance is also responsible for the health of the environment. Environmental finance theory emphasizes the innovation of financial technology, the upgrading of financial products, and the rationality of financial structure, which provides effective financial support for environmental protection. At the same time, environmental finance theory also involves some deep-seated institutional arrangements.

On the one hand, compared with traditional finance, the digital operation of finance can effectively reduce the consumption of resources, improving the efficiency of resource allocation, which shows an obvious “green effect” on finance and its related industries. On the other hand, digital finance can expand the coverage of financial services, guide and encourage more financial resources to low carbon, environmental protection, technological innovation, and high-tech industries. At the same time, it effectively curbs pollution investment, which not only helps to accelerate the green transformation of China’s economy, but also helps to promote technological progress in environmental protection, new energy, and energy conservation. In addition, digital finance can play the role of lubricant, accelerate the free circulation and effective allocation of capital, information, digital, technology, and other elements, and correct the market failure and financial fragmentation caused by information asymmetry in traditional finance [42,43,44]. It is an important hand to realize green development, transformation, and upgrading, and plays a decisive role in supporting green industry and sustainable economic and social development. Based on the above analyses, we propose the first research hypothesis.

**Hypothesis 1.**  *Digital finance is conducive to promoting green development.*

### 3.2. The Spatial Effect of Digital Finance on Green Development

According to the first law of geography, digital finance, as an economic phenomenon, inevitably has spatial correlation, which is significantly affected by geographical distance. Furthermore, through the theory of space economy and the theory of factor flow, it can be found that digital finance may release this spillover effect through the flow of various financial elements between regions.

The development of digital finance cannot be separated from traditional finance and the real economy, and the real economy cannot be separated from data, talent, technology, and other production factors. Therefore, the emergence and development of digital finance first appeared in metropolises with a high level of economic development, rich financial elements, and complete mobile Internet facilities. Since then, as “Digital China” has become a national strategy, the degree of financial marketization has been continuously improved, and the cross-regional flow of financial elements has made spatial correlation and interaction dependence increasingly strengthened. Driven by its own development needs and the profit-seeking characteristics of capital, digital finance began to expand and extend to other regions, forming a spatial inclusion. From this perspective, digital finance is not only conducive to local green development but also may have a “green spillover effect” on surrounding areas [45]. However, China’s financial market is still in the process of integration. Influenced by local governments, markets, and industries, the problem of regional segmentation is still obvious, and a unified financial market has not yet been formed. Although compared with traditional finance, digital finance has a wider coverage and higher liquidity, but in the case of insufficient high-level coordination, rigid constraints of administrative divisions, and local governments’ consideration of maximizing the interests within their jurisdiction, digital finance, as a resource, will also show scarcity among regions. Its development direction is mainly to meet the actual needs of local green development, while for other regions, it may inhibit the green spillover effect and even lead to potential pollution risks in surrounding areas. Based on the above analyses, we propose the second research hypothesis.

**Hypothesis 2.**  *There may be a spatial spillover effect of digital finance on green development, but this effect is uncertain.*

### 3.3. The Mechanism of Digital Finance Affecting Green Development

Under the guidance of sustainable development theory, green growth theory, energy economy theory, endogenous economic growth theory, and industrial structure upgrading theory, combined with existing research results, we believe that digital finance may affect green development from three paths: energy structure, technological progress, and industrial upgrading [46,47]. Of course, there should be many paths for digital finance to affect green development, which will be one of the key research contents and directions in the future.

According to energy economics theory, the relationship between energy utilization and environmental pollution has always been the focus of energy economics research. In China, energy utilization is an important source of environmental pollution and has a direct and widespread impact on green development. At the same time, as an advanced form of traditional finance, digital finance will inevitably enter the field of energy production and consumption, which will certainly have an important impact on the energy structure. Therefore, according to the guidance of relevant theories, we believe that digital finance may have an impact on green development by improving the energy structure. In addition, under the guidance of the “dual carbon” goal, green and low-carbon development is a complex project and a long-term task for sustainable economic and social development. Therefore, we must look for the direction and path to promote green development based on the basic national conditions of coal as the main fuel for a long time.

Specifically, digital finance can guide the reduction and replacement of backward production capacity in the energy industry, reduce energy consumption and gradually realize the transformation of energy structure through more green, environmentally friendly, and efficient technical support. At the same time, in the process of formulating and designing policies, products, and services, digital finance pays more attention to the orderly conversion of traditional energy to new energy and takes overall consideration to reducing the proportion of coal power and increasing the proportion of clean energy such as hydropower, wind power and photoelectric. Digital finance can play a further role as a strategy and tool for low-carbon transformation. With the help of structural monetary policy, diversified tools such as credit, bonds, equity investment, and trust can be used to provide financial support for green and low-carbon development. Therefore, digital finance can promote the adjustment of energy structure and gradually transition to green, low-carbon, and clean energy through technical support, capital allocation, and monetary policy for energy and related industries [48,49,50,51].

From the perspective of endogenous growth theory, combined with Solo models, sustainable economic growth comes from the improvement of total factor productivity, which is usually caused by technological progress [52]. As an advanced form of traditional financial integration with digital information technology, digital finance itself represents technological progress. At the same time, digital finance will also promote sustainable economic development through technological progress, which implies green development. In addition, from the perspective of industrial structure theory, the upgrading of industrial structure will help to improve productivity, reduce resource consumption, and reduce pollution and damage to the environment [53,54]. Therefore, it will help to achieve green development. Under the guidance of relevant theories, we believe that digital finance may promote green development through industrial upgrading and technological progress. In addition, from the perspective of externality theory, due to its significant positive externalities, digital finance can be actively encouraged by policies as an effective means of internalizing externalities, such as reducing the accuracy of orientation, rediscount, and refinancing, thus promoting green industrialization and industrial greening.

In addition to financial policies, the huge advantages of digital finance can not only provide targeted financial support for enterprises’ low-carbon transformation or green technology development, but also promote the development of the environmental protection industry, effectively overcome financing constraints, information asymmetry, moral hazard, and other problems, and provide financial security for enterprises’ green transformation. At the same time, financial policies can effectively curb the financing scale of environmentally unfriendly enterprises, increase the pressure on enterprise development, and ultimately promote enterprise technology upgrading and green transformation. In addition, compared with traditional finance, digital finance can effectively disperse the investment risk of enterprises, effectively gather and guide the green investment of social funds, and provide risk guarantee for enterprises’ green technology upgrading, green product development, and research and development of major energy conservation and environmental protection projects. Finally, digital finance can also provide the precise impetus for the low-carbon transformation of economic and social development by enabling green finance [42,43]. Based on the above analyses, we propose a third research hypothesis.

**Hypothesis 3.**  *Digital finance can boost green development by improving energy structure, promoting industrial upgrading and technological progress through the play of its own advantages.*

## 4. Model Constructions and Variable Selections

### 4.1. Model Constructions

We established a panel econometric model to effectively identify the possible impact of digital finance on green development. Hausman Test found that the fixed effect model is better than that of the random effect model, and hence, we chose the dual fixed effect model which controlled spatiotemporal effects. The model is constructed as follows:

(1) $$\ln gd_{it}=\alpha +\gamma \ln df_{it}+\phi X^{\prime }{}_{it}+a_{i}+\lambda_{t}+u_{it}$$

In the above formula, *gd* represents green development, characterized by energy consumption per unit of GDP; *df* is the digital financial index; $X^{\prime }{}_{it}$ are the control variables; ϕ is the coefficient of each control variable. $a_{i}$ and $\lambda_{t}$ represent individual and time effects respectively. γ is a constant term, and $u_{it}$ is the random error term.

Considering that there may be a spatial correlation between variables, the original assumption that samples are independent of each other is challenged, which may lead to biased error in the estimation of the general panel econometric model. Therefore, the spatial econometric model which takes spatial relationships into measurement is further constructed to capture more accurate causality between variables [55]. Taking the Spatial Durbin Model (SDM) as an example, the construction is as follows:

(2) $$\ln gd_{it}=\alpha_{1}+\rho W\ln gd_{it}+\gamma_{1}\ln df_{it}+\xi W\ln df_{it}+\phi X^{\prime }{}_{it}+\zeta WX^{\prime }{}_{it}+a_{i}+\lambda_{t}+u_{it}$$

In the above formula, W represents the spatial weights matrix. Considering the possible law of distance attenuation, we selected two kinds of matrices: spatial contiguity matrix and nearest neighbors matrix. $W\ln gd_{it}$, $W\ln df_{it}$ and $WX^{\prime }{}_{it}$ are spatial lag terms respectively, and ρ, ξ, ζ are coefficients of them.

Combined with the above Hypothesis 3, the mechanism of digital finance affecting green development is also one of the key research contents of this paper. Therefore, referring to the relevant research progress of Zhonglin Wen et al., we used the causal steps approach to empirically test whether there is a real mediation effect in energy structure, industrial upgrading, and technological progress [56,57]. The model is constructed as follows:

(3) $$\ln gd_{it}=\alpha_{2}+\gamma \ln df_{it}+\phi X^{\prime }{}_{it}+a_{i}+\lambda_{t}+u_{it}$$

(4) $$\ln mediator_{it}=\beta +\gamma_{2}\ln df_{it}+\phi X^{\prime }{}_{it}+a_{i}+\lambda_{t}+u_{it}$$

(5) $$\ln gd_{it}=\delta +\gamma_{3}\ln df_{it}+\eta \ln mediator_{it}+\phi X^{\prime }{}_{it}+a_{i}+\lambda_{t}+u_{it}$$

Three econometric models are needed to test the intermediary effect. Mediator variables represent intervening variables, which are energy structure, industrial upgrading, and technological progress. The remaining variables are consistent with those in formula (1). The verification steps are as follows:

Firstly, we should check $\gamma_{}$, $\gamma_{2}$, and η in the model (3)~(5). If the three coefficients are significant, it shows that there is a mediation effect. Secondly, we should check $\gamma_{3}$ in the model (5). If significant, it means that there is a direct effect, that is, “partial mediator”. Otherwise, only the mediation effect can be established. Thirdly, we should compare the signs of $\gamma_{3}$ and $\gamma_{2}\times \eta$. If the two are the same, it means that a partial mediation effect is established, otherwise, it is the hiding effect. Lastly, by comparison, we found that $\gamma_{3}$ in model (5) is lower than $\gamma_{}$ in model (3), which further indicates that the mediating variable plays a significant mediating role.

### 4.2. Variable Selections

(1) Explained variable. According to the energy economy theory and relevant research literature, we finally chose energy consumption per unit of GDP (*gd*) to represent green development, and the unit is metric ton standard coal/ten thousand CNY. Energy consumption per unit GDP can not only directly reflect the dependence of economic development on energy, but can also indirectly reflect the industrial structure and technological innovation level. The higher the energy consumption per unit GDP is, the stronger the dependence of economic development on energy is, which will be detrimental to reducing pollutant emissions and achieving green development. At the same time, high energy consumption per unit GDP also means a backward industrial structure and a low level of technological innovation. The high energy consumption per unit GDP reflects that the industrial structure is relatively backward, and the technological innovation level needs to be improved, which further reflects the low level of green development. Therefore, we agree it is reasonable to choose energy consumption per unit GDP as the indicator of green development. What is worth noting is that the energy consumption is calculated as regional comprehensive terminal energy consumption by four categories of coal, oil, natural gas and electricity, heat, and other energy. The reason why this paper picked terminal energy consumption is that it can truly reflect the actual energy consumption of economic and social development and people’s life.

(2) Core explanatory variable. We picked the digital inclusive finance index (*df*) published by Peking University Internet Finance Research Center over the years as the proxy variable of digital finance. This index is synthesized since financial service data provided by Ant Financial includes three dimensions: breadth of coverage, depth of use, and digitalization, which can systematically and comprehensively measure the development degree of digital finance [58].

(3) Mediator variables. According to Hypothesis 3, energy structure, industrial upgrading, and technological progress are three mediator variables. Among them, the energy structure is represented by the proportion of coal consumption (*cp*), and the reduction of the proportion of coal consumption will help promote the green development of the region. Industrial upgrading (*is*) referring to Linghui Fu [59], is represented by the advanced index of industrial structure calculated by the cosine method. Technological progress (*ti*) is represented by the expenditure on science and technology in fiscal expenditure.

(4) Control variables. To effectively avoid the possible estimation bias caused by omitted variables and to control as many factors as possible that affect green development, the following control variables are selected from the aspects of industrial structure, opening to the outside world, environmental regulation, transportation, etc. by referring to relevant theories and references. Industrial structure (*scbz*), measured by the proportion of tertiary industry; population density (*rkmd*), by the ratio of resident population to administrative area; opening to the outside world (*wstz*), by foreign direct investment; ecological environment (*ldmj*), by regional green space area; environmental regulation (*hbzc*), by environmental protection expenditure in fiscal expenditure; transportation (*rjgllc*), measured by road mileage per capita.

### 4.3. Data Sources

This paper takes 30 provincial-level units in China as samples, the Tibet, Hong Kong, Macao, and Taiwan excluded due to the lack of data. Green development mainly comes from the China Statistical Yearbook (http://www.stats.gov.cn/tjsj/ndsj/, accessed on 26 June 2022), the China Energy Statistical Yearbook, and regional energy balance tables (https://data.cnki.net/yearbook/Single/N2022060061, accessed on 6 July 2022). The digital financial index comes from Peking University Internet Finance Research Center (https://idf.pku.edu.cn/, accessed on 13 August 2022). The remaining economic and social statistics mainly come from statistical yearbooks and statistical bulletins of various provinces, autonomous regions, and municipalities. The air pollution data of robustness test are obtained from the online monitoring and analysis platform of China air quality (https://www.aqistudy.cn/, accessed on 26 August 2022). In addition, regarding the research period, taking full account of the accessibility, coherence, and authority of each variable data, the research period is limited to 2013–2020.

## 5. The Characteristics of Digital Finance and Green Development

### 5.1. Spatial and Temporal Characteristics

**Figure 2:**
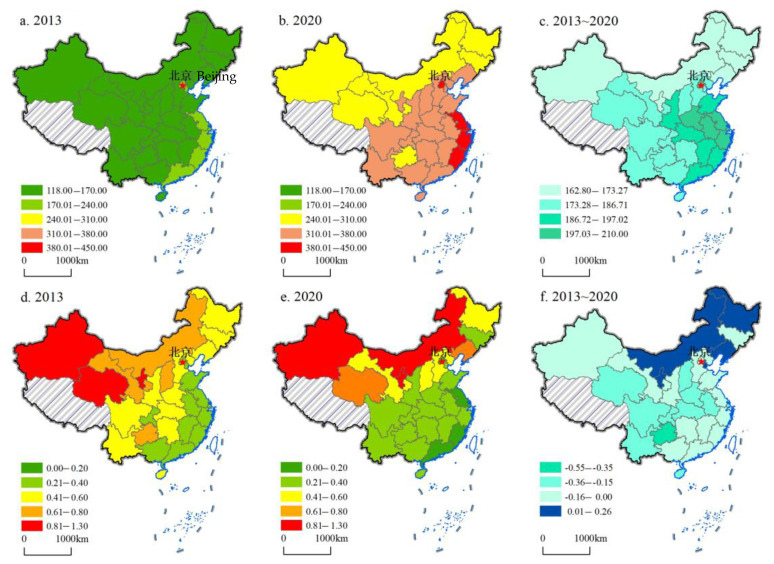
The spatiotemporal pattern characteristics of digital finance and green development. (**a**–**c**) represents digital finance, and (**d**–**f**) represents green development.

To have a comprehensive and systematic understanding of the current situation of digital finance and green development, 2013 and 2020 were selected to analyze the evolution of the spatiotemporal pattern with the help of the natural discontinuity point grading method. In the process of data analysis, we particularly emphasize the visual expression of data to obtain comprehensive spatial analysis results. The results are shown in Figure 2.

From the perspective of digital finance, the units with high levels in 2013 are mainly concentrated in the eastern region, such as Shanghai, Beijing, Zhejiang, Guangdong, Fujian, etc., while the level of digital finance in the central and western regions is relatively low, such as Qinghai, Guizhou, Gansu, Ningxia, Yunnan, etc. By 2020, the overall spatial pattern has not changed, but the digital finance development level of all provinces, regions, and municipalities has been significantly improved, especially the units in the eastern and central regions, such as Shanghai, Beijing, Jiangsu, Anhui, Henan, Jiangxi, etc. Among them, Shanghai’s digital finance development level is the highest and the improvement is the largest, rising from 222.14 in 2013 to 431.93, while the level of digital finance in Inner Mongolia, Heilongjiang, Xinjiang, Liaoning, Jilin, etc. is low, and the promotion is slow, with a small growth rate. The characteristics of this time-space pattern indicate that the level of economic and social development is the basis for the development of digital finance. A higher level of economic and social development can effectively promote the rapid development of digital finance. In contrast, in the backward areas of economic and social development, digital finance lacks the impetus for development, which inevitably leads to a low level of development.

Green development and digital finance have similar spatial characteristics. As the green development level is represented by energy consumption per unit of GDP, it is a negative indicator. Therefore, it can be considered that the southeast region has low energy consumption per unit of GDP and a high green development level, while the northwest region has high energy consumption per unit of GDP and a low green development level. In 2013, Beijing, Jiangsu, Guangdong, Zhejiang, Fujian, and other provinces and cities led the country in green development, while Ningxia, Qinghai, Xinjiang, Guizhou, Shanxi, etc. obviously lagged. In 2020, the overall pattern did not change, but the energy consumption per unit of GDP of most provinces, autonomous regions, and municipalities decreased significantly, indicating that the level of green development is constantly improving. Among them, Guizhou, Shanxi, Sichuan, Hubei, Qinghai, and other central and western provinces, autonomous regions, and municipalities showed outstanding performance. It is worth noting that some provincial units did not show a good trend in green development. The energy consumption per unit of GDP did not decrease but increased, such as in Inner Mongolia, Ningxia, Liaoning, Heilongjiang, and Tianjin. Therefore, these provinces, autonomous regions, and municipalities still have great space and potential in saving energy and reducing consumption for realizing green transformation. Overall, the southeast of China is an economically developed area with a high level of production technology. Although it has a large energy consumption, compared with GDP, the energy consumption per unit GDP is low. However, the northwest and northeast regions are backward in economic development and low in production technology. Although the energy consumption is small, the unit GDP is still high.

### 5.2. Spatial Trend Analysis

**Figure 3:**
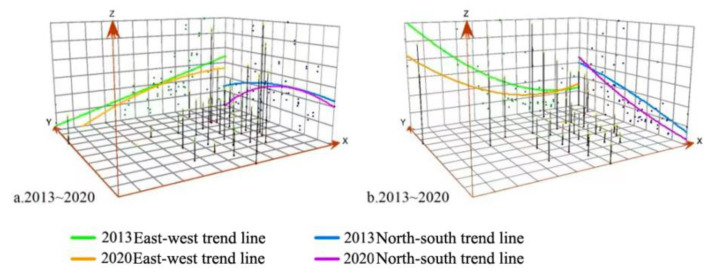
Analysis of the spatial trend of digital finance and green development.

To deeply explore the spatial change trend of digital finance and green development in the two directions of “east-west” and “north-south”, a trend analysis was conducted by ArcGIS10.6. Figure 3a represents the spatial change trend of digital finance, while Figure 3b represents the spatial change trend of green development. The *x*-axis represents the east-west direction, the *y*-axis represents the north-south direction, and the *z*-axis represents the level of digital finance or green development. We used the spatial trend projection line for analysis. Results are shown in Figure 3.

We can see from Figure 3a that, in 2013, the east-west projection trend line shows that the development level of digital finance gradually decreases from east to west and has a linear trend. The north-south projection trend line shows that the development level of the south is high and that of the north is low. By 2020, the trend lines in both directions begin to show a downward U-shaped trend, especially in the north-south direction. The change of this trend mainly lies in the obvious improvement in the digital financial level of some provinces, autonomous regions, and municipalities in the east and central China. It directly increases the projection value of the middle position. Looking from Figure 3b, in 2013, the east-west projected trend line indicates that the western region had high energy consumption per unit of GDP and low green development level, while the opposite is true in the east and middle. From south to north, the energy consumption per unit of GDP in the north is high and the level of green development is low, while the opposite is true in the south. By 2020, the overall trend of east-west and north-south directions does not change significantly, but the location of the projected trend line is closer to the *z*-axis due to the general reduction of energy consumption per unit of GDP.

## 6. Empirical Tests

### 6.1. The Benchmark Regression Results

Table 1 reports the regression results of the effect of digital finance on green development. Columns (1)~(2) belong to mixed cross-section regression, and columns (3)~(6) belong to dual fixed effect regression. To ensure the robustness of the results, the models without and with control variables are estimated separately.

The results showed that the causal relationship between digital finance and green development maintains a good negative robustness in different estimation models, and digital finance helps to significantly reduce energy consumption per unit of GDP, thus promoting green development. Therefore, digital finance has “green attributes” for economic development, which verifies Hypothesis 1. By gradually adding control variables, it is found that the estimated coefficient of digital finance on green development is stable at about 0.6, that is, if the development level of digital finance increases by 1%, the energy consumption per unit GDP will decrease by 0.6% at least. The reason for this result can be considered that digital finance has effectively reduced the carbon emissions of the financial industry and its related industries, improved the carbon emission efficiency, and has important significance and value for green development.

### 6.2. Analysis of Heterogeneity

Due to the vast territory of China, there are great differences in digitalization process, industrial structure, energy utilization, and economic volume among different regions. Therefore, digital finance may have heterogeneous effects on green development. Based on this, this paper tried to analyze it from different types, different regions, and the data structure itself. The results are shown in Table 2.

**Figure 4:**
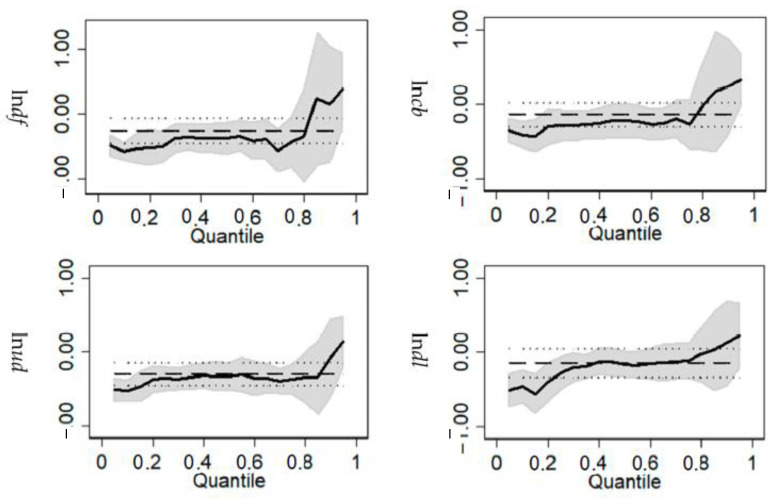
Quantile regression results of the impact of digital finance on green development.

(1) Digital finance has multi-dimensional and diversified characteristics. To systematically study the impact of digital finance on green development, it is divided into three dimensions: breadth of coverage (*cb*), depth of use (*ud*), and degree of digitalization (*dl*). Columns (1)~(3) showed that all three dimensions of digital finance can reduce energy consumption per unit of GDP to varying degrees, thus boosting green development. The magnitude of impact from high to low is the degree of digitization, breadth of coverage, and depth of use. The reason for this result is that the degree of digitalization is the basis of digital finance. The level of digitalization directly affects and determines the development level of digital finance. Therefore, the degree of digitalization is an important factor affecting the role of digital finance. In contrast, coverage and depth of use are based on the degree of digitization, so they are less effective than the degree of digitization. Thus, it can be seen that the development of digital finance requires not only the expansion of coverage and the realization of in-depth excavation, but also, more importantly, digital financial infrastructure and the digitalization level of relevant supporting software and hardware.

(2) According to the differences in human geography, sub-regional tests were conducted from the east, central and western regions, as well as the south and the north. Columns (4)~(5) are listed as the subsample estimation results of the eastern and central and western regions, and it is found that the impact of digital finance on green development is slightly stronger in the eastern region than in the central and western regions. The reason may be that digital finance in the eastern region is relatively high and the digital financial service system is relatively complete, which can provide more comprehensive and detailed green financial services for economic development, thus producing a more significant green growth effect. In addition, Columns (6)~(7) listed the estimation results of subsamples in the north and south, and the results showed that the role of digital finance in green development in the north is greater than that in the south. The possible reasons lie in the high proportion of industry, especially heavy industry in the north, where the energy consumption is higher than in the south. Therefore, the development of digital finance plays a more significant role in reducing energy consumption per unit of GDP and improving the level of green development in the north.

(3) To comprehensively describe the conditional distribution trend of digital finance to green development from the data structure itself, with reference to Wei Guo [60], the quantile regression method is introduced. By using the self-help method, we construct a covariance matrix to estimate models from 0.1 quantile to 0.9 quantile, which includes digital finance and its breadth of coverage, depth of use, and degree of digitization. The results are shown in Figure 4. Overall, digital finance and its three dimensions have a significant negative impact on energy consumption per unit of GDP, which is consistent with the estimated conclusions above. However, it is worth noting that the quantile regression coefficients of digital finance on green development begin to increase after 0.8 quantile, and the influence of the right end of the conditional distribution is greater than that of the middle and left. It was indicated that in a few areas with a higher level of green development, digital finance does not show a significant effect on reducing energy consumption. That is, digital finance will play a more prominent role in areas with a low level of green development, which fully reflects the universal characteristics of inclusive growth of digital finance. The possible reason for this result is that regions with high levels of digital finance development are often also regions with advanced economic and social development. These regions have advanced production technology and experience, and strict environmental regulations. Therefore, the role of digital finance in reducing consumption in these regions is relatively small. In contrast, regions with low levels of digital finance development are often backward regions, which do not have high production technology and environmental regulation. Therefore, the consumption reduction effect of digital finance is more obvious.

### 6.3. Analysis of Spatial Effects

Table 3 showed the results of spatial correlation tests and spatial econometric model selections. Firstly, we measured the Global Moran’s *I* index of digital finance and green development separately. The results showed that both passed the significance test each year, indicating that both have significant positive spatial correlations, which creates conditions for the use of spatial econometric models to estimate the spillover effect of digital finance on green development. Secondly, we selected the spatial econometric models. Lagrange Multiplier Test and Robust Lagrange Multiplier Test showed that the spatial lag model has better adaptability than the spatial error model. The Likelihood-ratio Test shows that the spatial Durbin model cannot be simplified to spatial lag and spatial error model, and the dual fixed effect is better than the time or individual fixed effect. Therefore, the Dubin model with dual fixed effects is chosen. The estimation results were shown in Table 4. We took the estimation results of the Dubin model under dual fixed effects as an example to illustrate in detail. Meanwhile, the results of the spatial lag and spatial error models were also put into the table to verify the robustness of the results. Each model uses two kinds of matrices, spatial contiguity weights, and nearest neighbors weights.

The results showed that the estimation results of the spatial econometric models considering spatial relationships were similar to those of the general panel model, and digital finance still shows a positive impact on green development by reducing energy consumption per unit of GDP. Moreover, the spatial lag terms of green development (W*lngd) all passed the significance test in both the spatial Dubin and spatial Lag models, indicating that the positive spillover effect of green development between regions is obvious, and the full play of this effect is conducive to the reduction of energy consumption and high-quality economic development for the entire area. However, the spatial lag term of digital finance (W*lndf) does not pass the significance test, indicating that the spillover effect of digital finance on green development is not obvious, that is, digital finance can effectively improve the level of green development in the local region, but does not show a significant effect on the green development of surrounding areas, which verifies Hypothesis 2. The possible reason for this result is that digital finance, as an advanced financial service, is also subject to the invisible influence of regional and urban boundaries. Especially in China, administrative divisions and the resulting market segmentation may limit the play of digital financial spillover effects. Therefore, how to effectively eliminate the regional differences in digital finance, accelerate the flow of financial elements, and improve the level of digital financial infrastructure will be the key issues to promote the effective play of digital financial spillovers.

### 6.4. Robustness and Endogeneity Tests

To improve the credibility of the empirical test results, this paper adopts three methods: replacing the core explanatory variables, replacing the explained variables, and simultaneously replacing the core explanatory variables and the explained variables. The results were shown in Table 5.

First, we selected the air quality index (*aqi*) as one of the explained variables. Air quality is closely related to energy consumption, which may produce various air pollutants, thus affecting the level of air quality. At the same time, air quality is also an important basis for evaluating the green and sustainable development of a regional economy. Column (1) showed that digital finance can significantly reduce the air quality index. Since the smaller the air quality index, the better it is, it can be considered that digital finance can help to enhance and improve air quality. Moreover, we also selected the Greenness Index of economic growth (*gi*) from the China Green Development Index Report published by Beijing Normal University as an alternative variable. The index emphasizes the green efficiency of industrial development and carries out a comprehensive evaluation by integrating various indices of the three industries. Column (2) showed that digital finance has a significant positive impact on the greenness of economic growth, indicating that it helps to improve the green efficiency of industrial development.

Firstly, digital economy (*de*) is used as the alternative variable of digital finance. Digital economy and digital finance have a close relationship, and they have a lot in common. Meanwhile, digital finance is an important part of the digital economy. Therefore, with reference to the research results of Tao Zhao [61], the development of the Internet is taken as the core index, and digital transaction indicators are added to construct a digital economic evaluation index system. Column (3) showed that the results of the digital economy on green development verified the conclusion of benchmark regression. Secondly, referring to relevant research results, we chose “the proportion of computer services and software employees” as the alternative variable of digital finance, which can reflect the development level of digital finance to a certain extent. The estimation results in column (4) were similar to those in column (3), which also indicates a significant impact on green development.

The third is to simultaneously replace the explanatory variable and the explained variable. In column (5), total energy consumption (*nyxf*) is taken as the explained variable, and Internet employees (*ie*) as the explanatory variable. In column (6), the greenness index of economic growth (*gi*) is taken as the explained variable, and Internet users (*iu*) as the explanatory variable. These replacement variables have a high correlation with the original variables, which can approximately reflect the causality. The estimation results all passed the significance test in varying degrees, which once again verified the positive effect of digital finance on green development.

Finally, to alleviate the endogenous problems caused by various reasons, we took the number of Internet users and digital finance, which lagged by one period respectively, as instrumental variables to conduct the two-stage least square method [62]. The number of Internet users can reflect the popularity of the Internet, which is highly compatible with the extensive coverage and in-depth digitalization of digital finance. Moreover, because the variable with one lag period is correlated with the current period variable, but not with the disturbance term in the predestined current period, the estimation bias caused by endogeneity can be alleviated. The results shown in columns (7) and (8) of Table 5 indicated that the green development effect of digital finance still holds. In addition, the Durbin-Wu-Hausman Test found the original hypothesis was obviously rejected and that all explanatory variables are exogenous, indicating the existence of endogenous explanatory variables. The statistical value is 9.45, and the *p* value is 0.002. Under-identification test (Kleibergen-Paap rk LM statistic) found the original hypothesis was obviously rejected. Weak identification test (Cragg-Donald Wald F statistic, Kleibergen-Paap Wald rk F statistic) showed that the critical value of statistics was greater than 15% or 10%, indicating that there was no weak correlation within the instrumental variables. Based on the above analysis, we believed that the selection of instrumental variables was effective and reliable.

## 7. Impact Mechanism Tests

Combined with the above mechanism analysis, digital finance may contribute to green development by improving the energy structure, promoting industrial upgrading, and technological progress. In the following, the mediation effect model was used to identify and judge by stepwise test, and the results were shown in Table 6.

From the perspective of energy consumption structure, column (1) showed that digital finance can significantly reduce the proportion of coal consumption to improve the energy consumption structure. In column (2), both digital finance and energy consumption were significant, and the direct effect was of the same sign as the mediating effect. Therefore, it can be concluded that energy structure is the mediating variable of digital finance affecting green development, and it belongs to the “partial mediation”. Columns (3) and (5) showed that digital finance can significantly promote industrial upgrading and technological progress. Further, columns (4) and (6) showed that industrial upgrading and technological progress can be used as effective mediating variables for digital finance to influence green development. Therefore, the above conclusions can effectively confirm hypothesis 3.

## 8. Conclusions

Taking 30 provincial-level units in China as samples, this paper systematically discusses the characteristics and mechanism of digital finance and green development by using the research methods of human geography and environmental economics and makes an empirical test. The main conclusions are as follows:

(1) From the perspective of spatial and temporal patterns, digital finance, and green development were promoted to different degrees during the study period, but the inter-provincial differences remained. Digital finance is developing rapidly, especially in eastern and central China. Meanwhile, with the reduction of energy consumption per unit of GDP, the level of green development also improves in general, and the performance of the central and western regions is outstanding. Yet the energy consumption per unit of GDP is rising in some provinces and cities. In addition, from the perspective of spatial trends, digital finance and green development have different trends in the east-west and north-south directions, and the overall performance is “high in the east, low in the west, high in the south, and low in the north”.

The reasons behind this phenomenon are that the eastern region has a high level of economic and social development and advanced industrial structure and production technology, which can not only effectively promote the development of digital finance, but also achieve a high level of green development. However, the economic and social development level in the central and western regions is relatively low, the industrial structure is relatively backward, the green production technology and capacity are limited, and the impetus for green development is insufficient. Yet overall, with the implementation of the “dual carbon” strategy, the upgrading of industrial structure and technological progress, digital finance, and green development will continue to develop.

(2) Through the estimation of the econometric models, we found that digital finance can reduce energy consumption per unit of GDP, showing a significant role in promoting green development, and still obtain robust results by replacing variables. At the same time, results were heterogeneous according to different types, different regions, and different levels. In addition, the green spillover effect of digital finance is not obvious, and its role is mainly concentrated in the local area. Furthermore, through mediation model testing, it is found that digital finance can achieve green development by improving energy structure, promoting industrial upgrading, and technological progress.

Through the above results, we can find that digital finance can indeed promote green development. The possible reasons are that, on the one hand, digital finance can effectively reduce the carbon emissions of the financial industry and its related industries by improving operational efficiency. On the other hand, digital finance can provide more inclusive and green financial services and products, which are conducive to industrial upgrading, technological progress, and green development of enterprises. Finally, the positive role of digital finance in factor flow, resource allocation, and efficiency improvement will also help to achieve green development.

## 9. Suggestions and Discussion

The conclusions of this paper are helpful to enrich and expand the theory of environmental finance. Generally speaking, the theory of environmental finance focuses on the impact of traditional financial services on environmental protection and advocates the responsibility for environmental quality. This paper extends the environmental finance theory from traditional finance to digital finance and provides theoretical support and empirical evidence for the impact of digital finance on green development. Therefore, this paper has certain theoretical implications. At the same time, this paper will also provide management implications for local government managers in developing digital finance and promoting green development. Therefore, based on the conclusion of the foregoing research and the actual development of our country, the government, financial institutions, and enterprise put forward the following countermeasures and suggestions for the mutual promotion of digital finance and green development:

Firstly, the government should give more attention to the role of encouragement and supervision. Focusing on the goal of “carbon emission peak and carbon neutrality” and the general requirements of green and low-carbon development in the economy and society, the government should strengthen the top-level design of digital finance, establish, and improve the laws, regulations, and policy system to promote green development. Further, we suggest the government enrich the toolbox of green finance support policies, coordinate and introduce more preferential policies to support green and low-carbon development, and guide financial institutions to promote industrial upgrades. Moreover, to comprehensively enhance the ability of digital finance to support green and low-carbon development, focusing on green technology innovation, increasing the allocation of green assets, and strengthening environmental risk management are also essential. In addition, the government should also accelerate the construction of the digital financial regulatory system, improve the laws, detailed rules, and regulations of green finance, and quickly improve the regulatory regulations according to the characteristics of digital finance to avoid the absence of regulation.

Secondly, financial institutions should play the leading role of digital finance in leading green development. On the one hand, we suggest that financial institutions follow the principle of “domestic unification and international integration”, focus on energy consumption, pollution control, energy conservation, emission reduction, green technology, and other fields, constantly improve the digital financial policy and standard system, so as to provide an important guarantee for standardizing digital financial business, ensuring the commercial sustainability of green finance and promoting the green development of economy and society. On the other hand, we suggest that financial institutions coordinate and develop specialized green financial products in different regions, design specific investment plans according to the R&D cycle of green technologies and the feedback effect of enterprises, vigorously promote business innovations such as green credit, green securities, green insurance, green guarantee, and green funds, etc. Moreover, they should also improve multi-level green financial products and a unified digital market system, provide more and better digital financial products and services for green and low-carbon development, and create the foundation and conditions for the green spillover effect.

Thirdly, from the perspective of enterprises, we suggest they could play the main role in realizing green transformation through digital finance. Enterprises are the main body of the application of digital financial products, but also the main position to achieve green transformation and development. Therefore, it is suggested to build a tripartite information-sharing platform and communication and cooperation mechanism of “government-bank-enterprise”, to smooth the interconnection of capital, technology, talents, and markets, and help enterprises better integrate into the capital chain, value chain, and industrial chain of digital finance. On the one hand, enterprises should make full use of digital financial products and platforms to improve production technology and promote green transformation and environmental benefits. On the other hand, enterprises should also pay attention to industry-research cooperation, set up green digital financial construction teams, and train professionals who understand not only financial knowledge but also digital and environmental protection technology, to lay a solid foundation for the high-quality development of green financial business.

Finally, this article basically achieved the set goals and completed tests of hypotheses. under the guidance of the environmental finance theory and related theories, and with reference to the latest research results, we selected provincial units in China as the research samples and obtained credible conclusions by selecting appropriate data and building models. However, we also realized that there are some deficiencies in this paper. The sample selection lacks attention to other administrative levels. For example, regional level, city level, enterprise level, etc. In addition, due to data availability and model design requirements, more possible factors were not considered when selecting intermediate variables. The impact of digital finance on green development is complex and diverse, which needs further analysis and verification. In the future, we think there are three warm tips for future researchers. First, the scope of digital finance can be extended to coverage, depth of use, and other aspects. Second, this paper reflects green development by using energy consumption per unit GDP. In future research, attention can be paid to the water environment, soil environment, air environment, etc. These are important indicators reflecting the level of green development. Third, we suggest that scholars explore how digital finance plays a role in green development at the enterprise level.

## Figures and Tables

**Table 1:** Benchmark regression results on the impact of digital finance on green development.

	Mixed Regression		Fixed Effect Panel Regression			
	(1)	(2)	(3)	(4)	(5)	(6)
ln*df*	−0.750 ***	−0.269 ***	−0.654 **	−0.785 ***	−0.748 ***	−0.627 **
	(0.093)	(0.099)	(0.259)	(0.257)	(0.249)	(0.248)
ln*scbz*		0.520 ***		0.513 ***	0.354 **	0.439 ***
		(0.156)		(0.160)	(0.161)	(0.161)
ln*rkmd*		−0.157 ***		−0.756 ***	−0.737 ***	−0.876 ***
		(0.032)		(0.273)	(0.267)	(0.295)
ln*wstz*		−0.147 ***			0.033	0.030
		(0.029)			(0.023)	(0.024)
ln*hbzc*		−0.195 ***			−0.147 ***	−0.142 ***
		(0.040)			(0.038)	(0.037)
ln*ldmj*		0.116 ***				−0.256 ***
		(0.036)				(0.081)
ln*rjgllc*		0.021				−0.122
		(0.069)				(0.161)
*_cons*	3.198 ***	2.396 ***	2.519 *	7.763 ***	7.801 ***	11.234 ***
	(0.509)	(0.620)	(1.305)	(1.905)	(1.850)	(2.290)
Fixed effect	uncontrol	uncontrol	control	control	control	control
*N*	240	240	240	240	240	240
*Within R^2^*	0.213	0.724	0.453	0.495	0.527	0.547

* *p* < 0.1, ** *p* < 0.05, *** *p* < 0.01. The significance level in the following table is the same as that in this table.

**Table 2:** Heterogeneity test of the impact of digital finance on green development.

	Classified Type			Classified Region			
	(1)	(2)	(3)	(4)	(5)	(6)	(7)
ln*cb*	−0.169 **						
	(0.067)						
ln*ud*		−0.111 **					
		(0.051)					
ln*dl*			−0.621 ***				
			(0.131)				
ln*df*				−1.252 **	−1.114 ***	−1.436 ***	−0.264 ***
				(0.482)	(0.368)	(0.365)	(0.087)
*X′*	control	control	control	control	control	control	control
Fixed effect	control	control	control	control	control	control	control
*N*	240	240	240	88	152	120	120
*Within R^2^*	0.594	0.591	0.656	0.778	0.700	0.505	0.889

** *p* < 0.05, *** *p* < 0.01.

**Table 3:** Spatial correlation and econometric model selection tests.

Spatial Correlation Tests			Model Selection Tests	
Year	Global Moran’s *I* of *gd*	Global Moran’s *I* of *df*	Test Methods	Statistical Values
2013	0.323 ***	0.247 ***	Hausman	21.79 ***
2014	0.322 ***	0.231 ***	LM-Spatial error	9.89 ***
2015	0.348 ***	0.216 **	Robust LM-Spatial error	0.07
2016	0.346 ***	0.225 ***	LM-Spatial lag	15.45 ***
2017	0.366 ***	0.277 ***	Robust LM-Spatial lag	5.63 ***
2018	0.361 ***	0.322 ***	LR-Spatial error	20.60 ***
2019	0.395 ***	0.318 ***	LR-Spatial lag	15.20 **
2020	0.397 ***	0.333 ***	LR-SDM-city	38.68 ***
			LR-SDM-time	432.62 ***

** *p* < 0.05, *** *p* < 0.01.

**Table 4:** Estimated results of the spatial econometrics for the digital finance and green development.

	Spatial Durbin Model		Spatial Error Model		Spatial Lag Model	
	(1)	(2)	(3)	(4)	(5)	(6)
ln*df*	−0.568 **	−0.400 *	−0.581 **	−0.535 **	−0.544 **	−0.568 ***
	(0.250)	(0.218)	(0.232)	(0.230)	(0.213)	(0.213)
*W*×ln*df*	0.117	0.132				
	(0.428)	(0.331)				
*W*×ln*gd*	0.211 **	0.220 ***			0.333 ***	0.337 ***
	(0.086)	(0.080)			(0.072)	(0.073)
λ			0.333 ***	0.399 ***		
			(0.083)	(0.083)		
*X′*	control	control	control	control	control	control
*W*×*X′*	control	control	control	control	control	control
Fixed effect	control	control	control	control	control	control
*N*	240	240	240	240	240	240
*Within R^2^*	0.615	0.670	0.575	0.574	0.570	0.564

* *p* < 0.1, ** *p* < 0.05, *** *p* < 0.01.

**Table 5:** The robustness and endogenous results of digital finance affecting green development.

	Explained Variables Replaced		Explanatory Variables Replaced		Simultaneously Replaced		Instrumental Variable One	Instrumental Variable Two
	(1)	(2)	(3)	(4)	(5)	(6)	(7)	(8)
ln*df*	−0.317 *	0.995 ***						
	(0.185)	(0.221)						
ln*de*			−0.244 ***					
			(0.062)					
ln*ie*				−0.117 *	−0.103 ***			
				(0.063)	(0.029)			
ln*iu*						0.362 ***	−3.485 ***	
						(0.116)	(1.281)	
L.ln*df*								−1.126 *
								(0.605)
*X′*	control	control	control	control	control	control	control	control
Fixed effect	control	control	control	control	control	control	control	control
*N*	240	209	240	240	210	210	240	240
*Within R* ^2^	0.737	0.885	0.611	0.623	0.700	0.878	0.569	0.631

* *p* < 0.1, *** *p* < 0.01.

**Table 6:** Test results of the mediation effect of digital finance on green development.

	Energy Structure	Green Development	Industrial Upgrading	Green Development	Technological Progress	Green Development
	(1)	(2)	(3)	(4)	(5)	(6)
ln*df*	−0.742 ***	−0.158 *	0.052 ***	−0.270 ***	0.455 ***	−0.202 **
	(0.167)	(0.087)	(0.006)	(0.088)	(0.167)	(0.084)
ln*cp*		0.168 ***				
		(0.035)				
ln*is*				−1.579 *		
				(0.939)		
ln*ti*						−0.178 ***
						(0.035)
*X′*	control	control	control	control	control	control
Fixed effect	control	control	control	control	control	control
*N*	240	240	240	240	240	240
*Within R* ^2^	0.468	0.642	0.792	0.607	0.664	0.647

* *p* < 0.1, ** *p* < 0.05, *** *p* < 0.01.

## Data Availability

This paper takes 30 provincial-level units in China as samples; Tibet, Hong Kong, Macao, and Taiwan are excluded due to the lack of data. The green development (energy consumption per unit of GDP) mainly comes from the China Statistical Yearbook, the China Energy Statistical Yearbook, and regional energy balance tables. Digital finance data come from Peking University Internet Finance Research Center. The remaining economic and social statistics mainly come from statistical yearbooks and statistical bulletins of various provinces, autonomous regions, and municipalities. The air pollution data of robustness tests are obtained from the online monitoring and analysis platform of China’s air quality.
